# Constraints and Prospects of Improving Cowpea Productivity to Ensure Food, Nutritional Security and Environmental Sustainability

**DOI:** 10.3389/fpls.2021.751731

**Published:** 2021-10-22

**Authors:** Olawale Israel Omomowo, Olubukola Oluranti Babalola

**Affiliations:** Food Security and Safety Niche Area, Faculty of Natural and Agricultural Sciences, North-West University, Mmabatho, South Africa

**Keywords:** cowpea productivity enhancement, indigenous legume, *Vigna unguiculata*, nutritious human food, the largest producer status, smart biotechnological approaches, protein-rich fodder for livestock

## Abstract

Providing safe and secure food for an increasing number of people globally is challenging. Coping with such a human population by merely applying the conventional agricultural production system has not proved to be agro-ecologically friendly; nor is it sustainable. Cowpea (*Vigna unguiculata* (L) Walp) is a multi-purpose legume. It consists of high-quality protein for human consumption, and it is rich in protein for livestock fodder. It enriches the soil in that it recycles nutrients through the fixation of nitrogen in association with nodulating bacteria. However, the productivity of this multi-functional, indigenous legume that is of great value to African smallholder farmers and the rural populace, and also to urban consumers and entrepreneurs, is limited. Because cowpea is of strategic importance in Africa, there is a need to improve on its productivity. Such endeavors in Africa are wrought with challenges that include drought, salinity, the excessive demand among farmers for synthetic chemicals, the repercussions of climate change, declining soil nutrients, microbial infestations, pest issues, and so forth. Nevertheless, giant strides have already been made and there have already been improvements in adopting sustainable and smart biotechnological approaches that are favorably influencing the production costs of cowpea and its availability. As such, the prospects for a leap in cowpea productivity in Africa and in the enhancement of its genetic gain are good. Potential and viable means for overcoming some of the above-mentioned production constraints would be to focus on the key cowpea producer nations in Africa and to encourage them to embrace biotechnological techniques in an integrated approach to enhance for sustainable productivity. This review highlights the spectrum of constraints that limit the cowpea yield, but looks ahead of the constraints and seeks a way forward to improve cowpea productivity in Africa. More importantly, this review investigates applications and insights concerning mechanisms of action for implementing eco-friendly biotechnological techniques, such as the deployment of bio inoculants, applying climate-smart agricultural (CSA) practices, agricultural conservation techniques, and multi-omics smart technology in the spheres of genomics, transcriptomics, proteomics, and metabolomics, for improving cowpea yields and productivity to achieve sustainable agro-ecosystems, and ensuring their stability.

## Introduction

With the world population expected to increase by up to 70% by 2050, the global community is faced with the constraint of providing safe and secure food supplies to an increasing number of people. The human population is projected to reach the 9.8 billion mark by 2030, bringing immense challenges in feeding the global populace (Tian et al., 2016; Pais et al., 2020). This will be a huge task, especially for the African continent, to handle in an era of climatic change and a growing population that will double by the year 2050 (Adedeji et al., 2020). Not only is the task of coping with this high human population growth rate in terms of the conventional agricultural production system daunting; it is also not environmentally/ecologically sustainable (Roell and Zurbriggen, 2020). In addition to the burgeoning human population, other factors that are posing threats to improvement in agricultural productivity include among others, climatic change, the loss of fertile agricultural land to urbanization, the challenges of phytopathogens and pests, abiotic challenges; high levels of salinity, and drought. Therefore, there is an urgent need to devise novel and workable solutions to achieve sustainable means of enhancing productivity in terms of agro-products and their nutritional composition.

Cowpea [*Vigna unguiculata* (L.) Walp.] is an annual leguminous crop that is grown throughout the world, but it is grown mainly in semiarid regions. Cowpea is a diploid, having 2*n*=2*x*=22, with the size of its genome consisting of approximately 620 million base pairs (Lonardi et al., 2019). In terms of its importance, this indigenous African legume is economically, nutritionally, and environmentally the foremost crop that serves as a source of essential human dietary nutrients and as, a means of providing fodder for livestock. It also presents with other multi-functional traits, including the maintenance of the soil – ecology balance through nitrogen fixation in that it facilitates a symbiosis with nodulating bacteria (Ravelombola et al., 2017). Cowpea is of strategic importance to Africa in terms of the large quantities that can be produced and is, therefore, an important component in the economy (Walker et al., 2016). Having originated in Africa, cowpea is now grown worldwide in 100 countries (Singh, 2014; Gonçalves et al., 2016). The cowpea yield in 2020 was estimated to be in the region of 9.8 million, while by 2030, the projected yield is expected to rise to 12.3millon tons (Boukar et al., 2016). Cowpea is indeed a multi-faceted crop, providing revenue for millions of smallholder farmers, as well as for traders who sell the nutritious grain. By providing essential protein, minerals, and vitamins, it serves in most African countries, as a means of balancing the diet, thereby providing a cheaper means for accessing the necessary dietary nutrients and for positively influencing the well-being and health of the populace. In addition, all of its components are valuable as nutrients (Gonçalves et al., 2016) – the leaves, pods, and seeds are nutritionally high in protein, with less fat, and are used extensively as the vegetable component in diets. In both the urban and rural settlements in most African countries, women generate income by trading in processed cowpea food and snacks. Cowpea is also important in livestock production, where the plant’s leaves and vines are dried and used as fodder/feed supplements in livestock husbandry. Cowpea is a key resource for a large number of people in the developing world, mainly in the arid/semi-arid tropical regions of the world (Muñoz-Amatriaín et al., 2017). Cowpea dry grain contains 23–32% protein and essential amino acids (Carvalho et al., 2017). Also, the green cowpea seeds, fresh and immature pods, and leaves contribute vegetable sources for human consumption (Gerrano et al., 2017, 2019). Its fresh leaves are used as vegetables, the haulms (cowpea pod walls, stems, and leaves) are used as livestock fodder, providing dietary nutrients for animals, and as income for the farmers (Kebede and Bekeko, 2020). Cowpea is highly prized as a source of food, for fodder in livestock feeds, and an important but cheaper means of improving and boosting soil fertility through biological nitrogen fixation. As important as it is in human nutrition, cowpea is equally useful in providing the necessary energy and protein in livestock production. More so, owing to its adaptation to different climatic conditions and its ability to grow in a less-fertile soil environment, it is highly appreciated as forage and a potential fodder crop for the future (Alemu et al., 2016). It is a key leguminous crop in the arid and tropical regions of Africa, Asia, and Latin America (Xiong et al., 2016). Cowpea is relished as a source of nutritious food and a variety of snacks that provide humans with cheaper proteins, thereby enhancing food security (Agbogidi and Egho, 2012; Muranaka et al., 2016). Cowpea is a vital source of beneficial micronutrients, proteins, amino acids, antioxidants, vitamins, and minerals, with immense therapeutic and nutritional security benefits (Jayathilake et al., 2018; Olabanji et al., 2018; Gondwe et al., 2019; Irondi et al., 2019; Owade et al., 2020). It is often used in mixed cropping systems to offer the multi-functional benefits of a nutritious grain, as a fodder crop, and as a means to improve soil fertility (Agza et al., 2012; Belay et al., 2017). Importantly, it is useful in agro-ecological conservation. It is used mainly as an inter-crop with other food crops to boost soil fertility and add nutrients to degraded soil through its nitrogen-fixing property (Rego et al., 2015). It is postulated that cowpea can fix about 337kgN. ha-1 (Yahaya, 2019). The average nitrogen addition/contribution to the soil during the cowpea growth and development phase is in the range of 40–80kgN. ha-1 and sometimes up to 200kgN. ha-1 (Meena et al., 2015). Also, it is useful as a cover crop or an erosion-preventing crop; it helps in suppressing weeds; and also aids in the retention of moisture (Das et al., 2018). Another key advantage of cowpea production is that when used as an inter-crop with other crops, it induces the growth of beneficial soil microorganisms and reduces the use of synthetic agrochemicals (Bukovsky-Reyes et al., 2019; Sun et al., 2019). In terms of importance, cowpea production contributes significantly to economic productivity and environmental sustainability in Africa (Martins et al., 2003; Olajide and Ilori, 2017; Ovalesha et al., 2017; Cardona-Ayala et al., 2020).

The main cowpea-producing countries of the world are in sub-Saharan Africa, that is the Sudano-Sahelian vegetation region (Boukar et al., 2019). Nigeria has the highest production output, followed by Niger and Burkina Faso, in that order. In terms of the metric ton production levels of cowpea grain, Nigeria is the largest producer in the world (FAO, 2020).

The productivity of cowpea in different countries differs in terms of the production output per area cultivated as highlighted in Table 1. However, despite all of the mentioned benefits of cowpea production in Africa in terms of the economies of scale, agri-food/nutritional benefits, and environmental stability influences, its productivity output is limited, and its status as an underutilized leguminous crop persists. The challenges militating against improved cowpea productivity in Africa include the following: climatic change and its adverse consequences on crop productivity include the issue of infrequent and erratic rainfall arising from, among others, drought and aridity issues, the decline in soil nutrients, the excessive use of synthetic chemicals, low-yielding seed cultivars, and infestations of pests and microbial pathogens (Rascovan et al., 2016; Afutu et al., 2017). Diverse strategies have been deployed by researchers in an attempt to breed cowpea for productivity enhancement. These strategies span through the initial selected germplasm collection from cowpea wild relatives and its natural population for desired genetic traits in order to create an improve cowpea genotypic varieties with agronomic traits and morphology through conventional hybridization and progeny cross-breeding techniques. These earlier breeding research techniques contributed to the development of many improved cowpea accession lines in the germplasm. However, significant barriers of improving cowpea varieties through the conventional breeding techniques like the challenges of intraspecific and interspecific crossing, genetic variation, genotype-by environment interaction, among others still persist. The advent of molecular tools such as RAPD, AFLP, ISSR, and other assisted marker selection genotypic breeding, was a milestone that led to genetic gains in cowpea productivity improvement. The advantages that are associated with this DNA molecular tools include: they are highly reproducible, cost-effective, and also it can deploy in the analysis of a large number of samples having genetic differences. Moving ahead, the advancement in molecular biology techniques that span genomics, proteomics, transcriptomics, and metabolomics, means cowpea-breeding research could now encompass assessing gene regulation and expression patterns for both abiotic and biotic resilient cultivars. These advance molecular technologies have been deployed to discern genotypic diversity existing in cowpea genome globally. Also, these advanced techniques have help cowpea breeders through genetic engineering to select desired gene traits and transfer across genetic barriers for cowpea improvement.

**Table 1:** Production output and productivity of cowpea by some selected countries in the world, excluding Brazil as (adapted from Faostat, 2020).

S/N	Country	Production in tons	Yield per hectare	Area harvested	Inference on production	Inference on productivity
1	Nigeria	2,606,912	9,137	2,853,097	1st	7th
2	Niger	2,376,727	4,035	5,889,677	2nd	18th
3	Burkina Faso	630,965	4,826	1,307,336	3rd	12th
4	Ghana	215,350	19,862	11,898	4th	2nd
5	Tanzania	202,865	4,096	30,366	5th	6th
6	Cameroon	185,832	4,043	258,898	6th	9th
7	Kenya	179,399	4,367	11,154	7th	10th
8	Mali	157,739	3,767	160,412	8th	11th
9	Myanmar	136,411	11,425	119,398	9th	4th
10	Sudan	104,667	2,678	333,638	10th	17th
11	Mozambique	89,356	5,545	284,451	11th	20th
12	Democratic Republic of Congo	72,726	4,432	95,803	12th	15th
13	Senegal	60,422	6,889	260,408	13th	19th
14	Malawi	42,456	13,515	159,345	14th	13th
15	United States	23,632	4,296	169,279	15th	1st
16	China	15,652	8,876	209,371	16th	5th
17	Madagascar	13,000	8,907	14,596	17th	8th
18	Uganda	12,439	9,750	208,059	18th	16th
19	Sri Lanka	11,180	11,770	9,499	19th	3rd
20	South Africa	4,871	10,360	15,108	20th	14th

In summary, diverse technological tools have been deployed by researchers for cowpea-breeding enhancement, spanning the past, the present and future prospects that include [markers systems, genetics maps, high-throughput genotyping, and quantitative trait loci (QTL) analysis]. In addition, mutation breeding, tissue culture, reverse genetics, clustered regularly interspaced short palindromic repeats (CRISPR) technologies are being apply for genetic gain in cowpea. In spite of this progress, major efforts are still needed for cowpea productivity improvement because cowpea plant is a diploid with a very narrow genetic makeup and also, it reproduces through means of self-pollination. Therefore, to overcome this gap, innovative research efforts that transverse different continents are still required toward breeding cowpea for enhanced productivity. For Africa to leverage its position as the foremost producer of this vital indigenous legume, the continent must look ahead at ways of improving productivity by closing the gaps in yield and by limiting the constraints to cowpea productivity in an agro-ecologically sustainable way. Therefore, this review highlights the constraints of cowpea production in Africa, and also gives an overview into the way these challenges can be circumvented through the deployment of smart biotechnological techniques/applications and insights concerning mechanisms of action for implementing eco-friendly biotechnological techniques, such as the deployment of bio inoculants, applying climate-smart agricultural (CSA) practices, agricultural conservation techniques, and multi-omics smart technology in the spheres of genomics, transcriptomics, proteomics, and metabolomics, for improving cowpea yields and productivity to achieve sustainable agro-ecosystems, and ensuring their stability.

## Production Constraints

The production of cowpea in Africa, the epicenter of this foremost indigenous legume, is carried out mainly by subsistence farmers. The production output of these smallholder farmers is limited by diverse constraints that lead to low agronomic yields/productivity. The average yield of cowpea in Africa is about 600kg/ha, which is still below its estimated optimum potential yield of 1,500–2,500kg/ha (Kamara et al., 2018). Numerous constraints limit the improvement of cowpea yield and productivity in Africa. These limiting factors can broadly be termed as abiotic/biotic stresses and climatic variations, and they have had a huge influence on the overall productivity of cowpea grains and fodder vegetables that are produced in the different cowpea-producing nations of the world, and particularly in Africa.

### Abiotic Stresses

#### Drought

Drought is a major challenge/constraint to achieving worldwide food security and production enhancement. Drought adversely affects plant growth at all developmental stages, impairing the morphology of the plant and the biochemical and physiological processes operating in the planted crops. These aspects subsequently affects, among others, the uptake of vital nutrients for plant growth and the ability of the seeds to germinate and of the plant to photosynthesize (Fahad et al., 2017; Lamaoui et al., 2018). Drought stress has negative consequences on the vitality and vigor of seeds and impairs seedling growth (Hatzig et al., 2018). The optimum growth/developmental stages in planted crops are adversely affected by drought, as observed in a decline in the rate of germination, seedling emergence and growth; impairments in vegetative growth, cell division and elongation; with mitotic processes also being affected (Farooq et al., 2009).

Drought stress can adversely affect the functioning of vital enzymes. Among other influences, the flowering stage of the plant could be negatively affected, as also the photosynthetic rate and the assimilate partitioning process. All these conditions eventually reduce the planted crop yields (Anjum et al., 2011).

Drought also impairs the proper functioning of the plant cell by producing oxidative damaging reactive species (ROS), which destroy plant lipids and proteins (You and Chan, 2015). Drought leads to adverse influences on the growth, development, and reproduction ability of planted cowpea, which limits the yield and productivity of the planted crops (Verbree et al., 2015; Daryanto et al., 2017; Ravelombola et al., 2018a).

Numerous studies have been done and are also on-going due to the enormity of drought stress challenges on cowpea productivity enhancement. In a study by Cui et al. (2020), they evaluated cowpea drought tolerance potentials at seedling stage. The experiment was done using a total of 36 cowpea-breeding lines in a completely randomized manner under drought stress conditions. Their results revealed that four (4) Arkansas cowpea breed lines are drought-tolerant, and they ranked better in terms of chlorophyll, healthiness and lodging score when compared to the other 32 genotypes. Therefore, these four cowpea breed lines could be further exploited in cowpea-breeding improvement. Also, in a study to highlight the constraining effects of drought stress on above-ground traits in cowpea plant, (Ravelombola et al., 2018a) assessed drought stress induced changes in 17 above ground traits in 30 cowpea genotypes at the seedling stage of growth for 28days. Their findings showed that cowpea genotypes PI293568, PI349674, and PI293469 are slow to wilting, better adapted to drought, while the other susceptible genotypes are fast to wilt, the chlorophyll content is lower, and they undergo senescence faster too. The three (3) cowpea drought-tolerant genotype could be exploited further for advanced breeding.

More so, in a comprehensive study of drought tolerance response in cowpea plant (Carvalho et al., 2019) used four (4) cowpea genotypes to determine their physiological, biochemical and molecular response under water-limiting stress conditions. The output from this study highlighted the importance of stomata conductance, photosynthetic parameters, compatible solutes like anthocyanin and proline, as well as increase in enzymatic activity of reactive oxygen species scavenging enzymes like catalase, superoxide dismutase, glutathione reductase, guaiacol peroxidase. This study also characterized the drought gene expression profile of the four cowpea genotypes. Thirteen drought related genes were profile, and some of the genes were expressed higher than others under drought stress. The hallmark of the study was that cowpea genotype Cp5051 was the most drought tolerant due to a higher expression of drought-tolerant marker genes *VuHsp17.7* and *VuCPRD14.*

#### Salinity

Soil salinity is a major abiotic constraint to plant productivity. Salinity adversely impacts the metabolic and physiological processes in plants. Statistical report stipulated that upward of over 45 million hectares of agricultural soil are affected by this problem and that climatic change, as well as current irrigation practices, will exacerbate this situation (Munns and Gilliham, 2015; Parihar et al., 2015).

In addition, salinity stress negatively influences the rate of plant growth. The adverse influence of salinity reduces the fresh and dry weight of plants, while other vegetative growth traits are also adversely impacted (El-Beltagi et al., 2013; Mohamed et al., 2018).

Salinity stress leads to extensive damage in the adductive capacity of planted crops. It reduces lipid peroxidation and leads to the production of destructive oxidation species (ROS), that in turn causes damage to the key plant biomolecules (Ghonaim et al., 2021).

Salinity stress ultimately reduces yields and the productivity of planted cowpea crops, thereby affecting the goal of achieving enhanced global food security (Da Silva Sá et al., 2017; Ravelombola et al., 2018b).

Research into the impact of salinity on cowpea has indicated that it impairs cowpea seed germination, its vigor, and growth (Zahedi et al., 2012; Mini et al., 2015).

In order to evaluate salinity stress impact on cowpea cultivars (Ravelombola et al., 2019), investigated a simple protocol that could be deployed to assess the response of 30 cowpea genotypes to salinity constraint at seedling growth stage in a greenhouse experiment that profile 14 above ground traits response to sodium chloride (NaCl)-induced salinity. The findings indicated that relative salinity tolerance (RST) of cowpea genotype PI255774, all the plants were completely dead, while PI582438 performed best and the leaves were all green and had higher chlorophyll content. The outcome of this study validated simple protocol of assessing chlorophyll content and leaf injury for assessing salinity at seedling stage in cowpea. Also, in a study to investigate further the utility of chlorophyll content as a means of assessing salinity tolerance in cowpea seedling over time, (Dong et al., 2019) investigated how 24 different cowpea genotypes responded to salinity induced stress by monitoring the chlorophyll changes over a period of 24days using a split-plot design. The results indicated the importance of genotype and the timing in relation to cowpea seedling response to salinity stress. Also, the chlorophyll content of the cowpea salt-tolerant cultivar was higher at day 24 of the experiment, while all the cowpea salt sensitive plant were dead at the end of the 24days. In addition, salinity induce stress could further predispose cowpea cultivars to viral infestation. In their study, (Varela et al., 2019) assessed the consequences of exposing a cowpea severe mosaic virus (CPSMV)-resistant genotype to salinity induced stress. The results signify that vital protein pathways were altered, and there was proliferation of the (CPSMV), leading to the cowpea genotype changing from resistant to susceptible.

#### Heavy Metals

Heavy metals pose serious environmental constraints for and can adversely impact on plants and humans when the former bioaccumulate in plants and ultimately reach human beings *via* the food chain (Sidhu, 2016).

Heavy metals pose environmental and public health threats when they are discharged as by-products of industrial processes in the form of effluents (Wuana and Okieimen, 2011). Heavy metals, such as cadmium (Cd), lead (Pb), arsenic (As), mercury (Hg), chromium (Cr), and antimony (Sb), affect plant productivity and plant yields.

Heavy metals adversely affect the metabolic processes of the plant during the course of its growth and development. Heavy metals negatively influence the germination of seeds, while the vegetative growth rate (leaf, shoot, and root) is also impaired. Plants are adversely affected by heavy metals, as in the case of various physiological and biochemical processes such as the rate of photosynthesis, the uptake of nutrients, vital enzymatic reactions, as well as in the case of emergence of ROS (Azevedo and Rodriguez, 2012; Tiwari and Lata, 2018).

Research reported by Asagba et al. (2019) detailed the impact of Nickel toxicity on cowpea germination and other biochemical parameters. The investigation on phytotoxicity of nickel at varying concentration on cowpea seedling growth rate, length, fresh weight, as well as Ca^2+^ ATPase activity was assessed. The results indicated toxic impact of this heavy metal on cowpea seedling agronomic and biochemical parameters.

Also, in a study by Ogunkunle et al. (2020) that applied co-inoculation of arbuscular mycorrhizal fungi (AMF) and nano-TiO_2_ to reduced oxidative stress and bioaccumulation of Cd in cowpea, it was reported that the total chlorophyll of the cowpea plant, as well as different reactive oxygen species enzymes were impacted negatively due to Cd induced toxicity.

#### Temperature Stress

As an abiotic stress factor, temperature in the case of low temperatures (chill stress) and high temperatures (heating stress) is a potential constraint in limiting the productivity and yield of planted crops globally. Temperature is a key abiotic parameter that influences the growth and development of plants (Hatfield and Prueger, 2015).

High temperatures limit the photosynthetic rate of the plant. The vegetative growth parameters and the metabolic activities of the plant are also adversely affected. Also, emergence, maturity/ripening, harvesting time (length of period/stage), and plant yield are affected (Prasad et al., 2008; Shah et al., 2011). Likewise, low-temperatures (chilling stress) adversely influence plant metabolic activity and negatively impact the growth/development of plants (Tian et al., 2011). Low temperatures (chilling stress) also negatively affect the germination rate, seedling emergence, and the vigor of the plant, so that the productivity of the plant is ultimately reduced (Abbas, 2012).

In a study on the impact of elevated temperature on the agronomic growth parameters and the nutritional status of cowpea at different growth phase, (Nevhulaudzi et al., 2020b) reported that there were differences in both the agronomic growth and nutritional parameters, and this is more pronounced at the flowering and pre-flowering stage.

#### Waterlogging Stress

Waterlogging stress affects the gaseous exchange in agricultural soil and negatively impacts crop productivity globally. It leads to an insufficient supply of oxygen to the plant roots and this in turn reduces the growth and development of the plant roots. It also leads to the inability of the plant to take up the necessary nutrients and nitrogen. Waterlogging affects the photosynthetic rate, reduces the vegetative agronomic growth rate of plants, leads to the senescence of leaves, and ultimately, negatively affects crop yield and productivity (Ren et al., 2014).

Higher or excessive soil water availability do not always favor cowpea growth. In a field study done in the Sudan Savanna zone taking genotype environment interaction into focus, (Iseki et al., 2021) reported that excess water can inhibit the nitrogen-fixing capability of cowpea and lower its productivity.

#### Climatic Change Stress

Climatic changes in weather, as denoted by among others variability or fluctuations in the prevailing temperatures, rainfall, and the volume of greenhouse gases, are potentially limiting factors on various agro-input variables and ultimately affect the productivity of planted crops on a global scale (Awoye et al., 2017; Kukal and Irmak, 2018; Hounnou et al., 2019).

Climatic changes also adversely threaten the agri-food system at all scales: globally, nationally, regionally, and locally (Ajadi et al., 2011).

Climatic change negatively impacts agri-food input and output production systems because it influences the biotic and abiotic parameters of agricultural production. Hence, it affects planted crop yields (Challinor et al., 2014).

Changes in climatic conditions affect the biochemical, physiological, and metabolic activities of plants; the photosynthetic rate is affected, as are factors such as plant growth and development, and the rate of transpiration; there is also an imbalance in the elimination of CO_2_, and a reduction in enzyme reactions; flowering may be affected, which could also lead to senescence (Srivastava et al., 2019). Predictive studies have forecast a reduction in food grain yields toward the later years of this current century (Pachauri et al., 2014), this hinging on expected extremes in global temperatures. Furthermore, most, if not all of the major food crops are adversely impacted by stress arising from heat at the different growth and developmental stages (Kaushal et al., 2016; Atlin et al., 2017). Global changes in climatic conditions have been found to adversely affect the health of humans, animal/livestock production, as well as planted crop productivity (Lesk et al., 2016; Mora et al., 2017).

In summary, many huge tasking constraints are militating against and slowing down the optimum yield production of cowpea in Africa. Some of these limiting challenges are highlighted in Table 2.

**Table 2:** Highlight of constraints limiting productivity enhancement of cowpea plant in major producing nations of the world.

Productivity constraint	Crop of interest	Bioactive roles of stressors	References
Biotic limitation involving Cowpea Severe Mosaic Virus	*Vigna unguiculata*	The chlorotic lesion, mosaic formation, and necrosis	Oliveira et al., 2020
Combine abiotic stressors of CO_2_, High temperature and UVB irradiation	*Vigna unguiculata*	Vegetative and reproductive growth stage impaired adversely	Singh et al., 2010
Drought stress	*Vigna unguiculata*	Reduction in vegetative biomass Photosynthesis, transpiration, and stomatal conductance	Cardona-Ayala et al., 2020
Abiotic limitation involving heavy metals (Chromium)	*Vigna unguiculata*	Adverse impact on nodulation and biological nitrogen fixation	Miranda et al., 2014
Biotic constraint caused by Legume Pod Borer (*Maruca vitrata* Fabricius) (LPB)	*Vigna unguiculata*	Complete crop failure due to feeding on all parts of cowpea	Sodedji et al., 2020
Biotic constraint caused by *Aplosporella hesperidica*	*Vigna unguiculata*	Adverse impact on cowpea leading to collar rot symptoms	Deepika et al., 2020a
Biotic constraint caused by *Fusarium equiseti*	Vigna unguiculata	Negative impact on cowpea resulting in root rot symptoms	Li et al., 2017
Biotic constraint caused by *Fusarium oxysporum*	*Vigna unguiculata*	Negative impact on cowpea resulting in stem and root rot symptoms	Shrestha et al., 2016b
Biotic constraint caused by *Fusarium proliferatum*	*Vigna unguiculata*	Negative impact on cowpea resulting in stem and dry root rot symptoms	Shrestha et al., 2016a
Biotic constraint caused by Singly and Interactive effects of cowpea mosaic viruses	Vigna unguiculata	Negative impact on Rhizobium nodulating ability	Taiwo et al., 2014
Biotic constraint caused by *Rhizoctonia solani*	*Vigna unguiculata*	Negative impact on cowpea resulting in collar rot and web blight symptoms	Vavilappalli and Celine, 2014
Biotic constraint caused by *Helminthosporium vignicola*	*Vigna unguiculata*	Negative impact on cowpea resulting in leaf spot disease symptoms	Sahoo and Beura, 2019
Biotic constraint caused by *Epicoccum nigrum*	*Vigna unguiculata*	Negative impact on cowpea resulting in leaf spot disease symptoms	Deepika et al., 2020b
The abiotic constraint of Drought on cowpea Landrace (A55)	*Vigna unguiculata*	Reduction in net productivity and photosynthetic ability	Gomes et al., 2020
The abiotic constraint of high temperature	*Vigna unguiculata*	Adverse impacts on physiology biochemistry and breeding traits in cowpea plant	Jha et al., 2020
Biotic constraint caused by *Dactuliophora mysorensis* sp. nov	*Vigna unguiculata*	Zonate leaf spot disease	Deepika et al., 2020c
Biotic constraint caused by *Nigrospora sphaerica*	*Vigna unguiculata*	Leaf spot disease	Deepika et al., 2021
The abiotic constraint of high salinity	*Vigna unguiculata*	Adverse impacts on chlorophyl content and eventual death	Dong et al., 2019
The abiotic constraint of high-temperature stress	*Vigna unguiculata*	Adverse impacts on plant development, with severe damage to vegetative and reproductive growth stages of cowpea	Barros et al., 2021
The abiotic constraint of combined high salinity and temperature stress	*Vigna unguiculata*	Adverse impacts on plant development, with the germination and vigor of cowpea plant, impaired	Nunes et al., 2019
Climate change limitation involving temperature and Relative humidity	*Vigna unguiculata*	Adverse impacts on the yield and development of cowpea plant as well as reduction in evapotranspiration	Cavalcante Junior et al., 2016
Biotic constraints caused by *Diplodia seratia*	*Vigna unguiculata*	Wilt and necrosis adverse effects on cowpea	Swilling et al., 2020

### Biotic Stress

Worldwide, biotic stressors (roots and membrane pathogens) in large numbers lead to low productivity and low-quality agricultural products. Destructive pests and pathogens result in food insecurity on every scale – from the smallest to the largest thus leading to massive monetary losses on a global scale in terms of crop yield (Savary et al., 2019).

The main production constraints concerning biotic stress factors limiting cowpea productivity are exemplified by a wide range of organisms, including destructive pests; parasitic weeds, viral pathogens, bacterial pathogens, as well as fungal pathogens (Boukar et al., 2019).

#### Bacterial Diseases/Pathogens Affecting Cowpea Seeds, Plants, and Pods

A major constraint in limiting cowpea yields can be attributed to bacterial pathogens, which lead to massive crop losses of upward of 70% in the form of seed grain, pod, and fodder reduction (Agbicodo et al., 2010). Some of these destructive pathogens are transmitted *via* the seed (De Lima-Primo et al., 2015), while some are transmitted *via* the soil-borne route (Constantin et al., 2016). Some of the damaging symptoms of bacterial pathogen infestation in cowpea are brownish leaf spots, necrotizing and yellow halo leaf shapes, cracks noticeable on the stem, and pods filled with water, and blotch (Claudius-Cole et al., 2014). Among the most destructive bacterial pathogens of cowpea are members of the *Xanthomonas* genus (Shi et al., 2016; Durojaye et al., 2019).

#### Root-Knot Nematodes

Nematodes are responsible for huge losses in cowpea production and are also one of the constraints limiting improvements in cowpea production (Haegeman et al., 2012; Dareus et al., 2021). This they accomplish by impeding the uptake of water and nutrients. Also, nematodes limit cowpea growth and development by interfering in the pathways towards cell differentiation and in the transportation of auxin (Gheysen and Mitchum, 2011). *Meloidogyne javanica* and *Meloidogyne incognita* are the two major nematodes destroying cowpea (Oliveira et al., 2012).

#### Fungal Diseases/Pathogens Associated With Cowpea

Fungal pathogens are the topmost destructive agents/phytopathogens of planted crops globally (Fisher et al., 2012). Very many species of different genera of fungi destroy cowpea in the field and during the post-harvest stage. Furthermore, seed and soil-borne fungal pathogens have been implicated in the loss of cowpea production that sometimes rises to 100% (Mbeyagala et al., 2014). Notable fungal pathogens of cowpea include *Rhizoctonia solani*, *Colletotrichum* spp., *Fusarium oxysporum*, *Macrophomina phaseolina*, and *Sclerotium rolfsii* (Adegbite and Amusa, 2010; Pottorff et al., 2014).

#### Viral Diseases/Pathogens Associated With Cowpea

Viral pathogens can adversely impact cowpea productivity; some of these have been linked in some cases to cowpea losses of up to 100% (Nsa and Kareem, 2015). Their destructive mechanisms that negatively affect cowpea include the reduction they cause in the population/growth and development of Rhizobium, thereby reducing the necessary root nodulation in cowpea (Taiwo et al., 2014). Up to 40 viruses adversely affect cowpea yields globally. Some of the most devastating viral pathogens of cowpea are the cowpea aphid-borne mosaic virus (CABMV), cowpea wild mottle virus (CPMMV), and CPSMV (Boukar et al., 2013; Odedara and Kumar, 2017).

#### Parasitic Weeds

**Figure 1:**
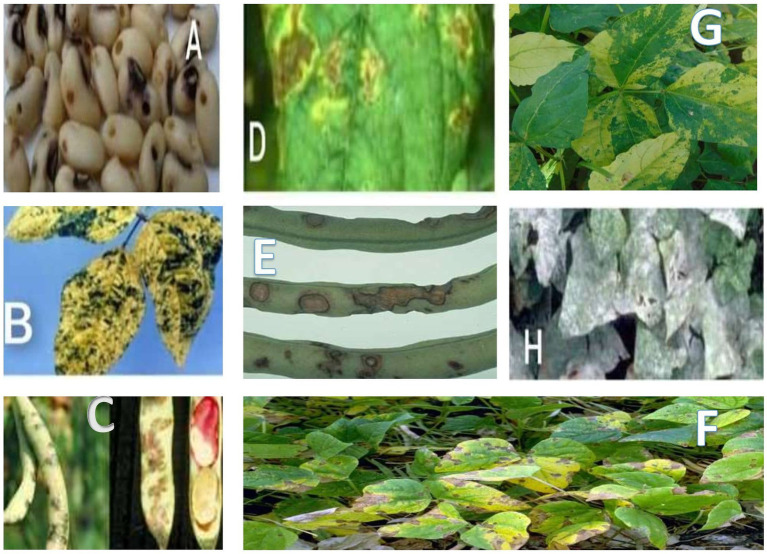
Microbial diseases of cowpea: **(A)** cowpea seed beetle, **(B)** yellow mosaic virus infected cowpea, **(C)** cowpea halo blight, **(D)** bacterial blight, **(E)** anthracnose, **(F)** cowpea mosaic diseased leaf, **(G)** bacterial bean blight, and **(H)** powdery mildew.

Parasitic weeds cause serious losses in cowpea production/yields (Li and Timko, 2009; Horn et al., 2015; Omoigui et al., 2017). Eliminating these weeds in the course of cowpea production is difficult because they could be dormant in the soil for upward of 20years (Kamara et al., 2014). The major parasitic weeds that adversely affect the enhancement of cowpea production in Africa are *Striga gesnerioides* and *Alectra vogelii* (Figure 1).

## Looking Ahead Beyond the Constraints for Cowpea Productivity Enhancement with Sustainable Bioinoculants and Smart Biotechnological Techniques

It is worth re-emphasizing that the challenge of attaining enhanced cowpea productivity on a sustainable level is not merely a single limitation. Rather, it is a diversity of limitations requiring a high level of multi-tasking.

However, there are also multiple smart, and sustainable agro-biotechnological techniques that could be deployed in a sustainable manner to achieve improvements in cowpea productivity and production outputs. Elements of this technology, which is geared towards maximizing eco-friendliness and guaranteeing an improvement in safer agro-biotechnological productivity, are briefly listed, and their associated mechanisms of action are also explained.

1. The sustainable deployment of bio-inoculants (biofertilizers and biostimulants) to serve as an alternative to synthetic chemicals
2. The sustainable deployment of biological antagonists in the form of biopesticides to tackle pests in the field and during the post-harvest storage stage
3. The deployment of CSA practices as an adaptive technology option to mitigate the effects of climate change on the vulnerabilities of crop production
4. The deployment of smart and advanced biotechnological applications, such as metabolomics, transcriptomics, proteomics, and genomic-breeding tools for the improvement of cowpea varieties, which would possess the desired traits, such as drought tolerance, favorable salinity stress-tolerant levels, high yields, resistance to high temperatures and thermotolerance, resistance to disease, and a high potential for nodulation.
5. The application of conservation practices in agriculture

### Sustainable Deployment of Bio-Based/Microbial Resources as Alternative to Synthetic Agrochemicals

Microbial-based formulations have proved to be an effective alternative to the use of synthetic agrochemicals in crop production. These natural, eco-friendly and sustainable bioformulants are categorized as biopesticides, biostimulants, and biofertilizers.

To minimize crop losses and improve productivity, natural microbial-based formulations have been successfully deployed in agro-ecological crop production. The salient features of these resources are that they are cheaper, renewable, easy to handle, and more importantly, safe for human beings and the living environment (Kour et al., 2020; Castaldi et al., 2021).

To meet up with the challenge of feeding the rapidly increasing global population, there is a need to increase crop productivity. One popular means of solving the problem of global food insecurity is by boosting agricultural outputs/productivity through the application of synthetic agro-fertilizers.

Conventionally, synthetic agrochemicals are applied as inputs to intensify agricultural production systems. Various fertilizers, fungicides, herbicides, and pesticides are thus used in large-scale crop production systems. Initially, the advent of the chemical fertilizer was widely accepted because it helps to increase agricultural productivity and to solve global food consumption issues (Liu et al., 2015; Duan et al., 2016). However, the indiscriminate use of chemical fertilizers has led to air and groundwater pollution, which, mainly in the case of the latter has led to the eutrophication of water bodies (Vanlauwe et al., 2014). Also, the long-term effect of using chemical fertilizers results in bio-magnification and bio-accumulation in living organisms which have in their turn had negative impacts on the soil environment and ultimately on human and animal health (Calderón et al., 2017).

Therefore, the increasing concern of consumers and governments for food safety issues, has led stakeholders to explore newer ecologically and environmentally-friendly methods to replace or supplement the current chemical-based practices in agriculture. In fact, the use of bio-pesticides, bio-herbicides, and bio-insecticides has emerged as a promising alternative to chemical pesticidal products (Ahirwar et al., 2020).

Also, (Nicolopoulou-Stamati et al., 2016) reported that the use of chemicals in the form of pesticides, insecticides, and herbicides could affect the quality of the plant products and thus adversely affect human and animal health.

However, the search for environmentally and agro-ecologically sustainable alternatives to these synthetic agrochemicals has led to the deployment of quite an array of diverse forms of microorganisms being applied to function as biofertilizers, biostimulants, biopesticides, and plant growth promoters. Hence, they are being used to enhance a diversity of crop growth in numerous countries around the world, especially in the developing and emerging world (Igiehon and Babalola, 2017; Alori and Babalola, 2018; Omomowo and Babalola, 2019).

Different groups of microorganisms constitute different types of association with different host plants in the form of endophytic, epiphytic, and rhizospheric associations (Yadav, 2021). Thus, based on these associations, scientists have formulated bio-inoculants to solve the food security problem in an eco-friendly way.

Diverse terminologies have been used to qualify these metabolically and physiologically important microbial forms. They are known under terms such as biocontrol agents (BCAs), and are referred to as agriculturally beneficial microorganisms, e.g., arbuscular mycorrhizal fungi (AMFs), which are sometimes referred to as, among others, plant growth-promoting rhizobacteria, plant growth-promoting fungi (PGPFs), and plant growth-promoting bacteria (PGPBs). A lot of research in the field of applying microbial inoculants to different planted crops has been conducted by scientists and is still ongoing (Igiehon et al., 2019; Chaudhary et al., 2021; Chen et al., 2021). These beneficial species help to control or suppress plant diseases caused by pathogenic bacteria and fungi through different antagonistic mechanisms in that they produce antifungal and antibacterial compounds or feed as parasites on them (El-Sharkawy et al., 2018).

To solve the problem of food safety and the increasing concerns in respect of the environment in an eco-friendly manner, the use of biofertilizers, biopesticides, and biostimulants is gaining the necessary attention in the agricultural sector (Oleńska et al., 2020). Based on plant-microbial associations, the utilization of viable and sustainable microbiota or their groupings has long been established as a means to improve agricultural productivity, and is in fact on an upward rise (Chukwuneme et al., 2020; Adeleke and Babalola, 2021; Fasusi et al., 2021).

More importantly, with the advent of next generation sequencing technological availability and cheaper cost, research efforts in the field of metagenomics, metabolomics, proteomics, transcriptomics and genomics have revolutionize the prospects of applying plant growth-promoting microbiota as bioinoculants that are deployed as biofertilizer, biopesticides and biostimulants for the improvement of planted crops. With the advent of these advanced biotechnological techniques, researchers have elucidated studies on the root microbiome as the hidden treasure that possesses immense potential to revolutionize the strategies for improving plant growth, as well as abating biotic and abiotic constraints in plants (Mathur and Roy, 2021).

These root-associated microbiomes are known as prolific producers of phytohormones, mainly auxins, cytokinin, and ethylene as well as enzymes like the 1-aminocyclopropane-1-carboxylate deaminase (ACC deaminase) and exopolysaccharides that help plants in inducing systemic resistance to both biotic and abiotic stressors. Newer and effective techniques have been deployed in isolating and characterizing root associated microbiome, and applying them as bioinoculants in improving the growth and development of planted crops (Liu et al., 2020; Romano et al., 2020).

The root microbiome consists of an enormous number of beneficial microbes such as plant growth-promoting rhizobacteria (PGPRs), fungal and bacterial endophytes and mycorrhizal fungi (Yu et al., 2019).

Metabolites that are secreted by this microbiota are associated with marked influences on plant growth promotion, response and mitigation to biotic and abiotic stressors. These bioactive metabolites include ACC deaminase, gibberellic acid (GAs), indole acetic acid (IAA), exopolysaccharides, melatonin, volatiles, and cytokinins (Jones et al., 2019; Qu et al., 2020).

It is anticipated that root exudates influence the rhizospheric microbial community and that analysis of the root microbiome signifies ecosystem functioning (Williams and de Vries, 2020). Therefore, a lot of research effort abound on exploration of the root microbiome as reservoir of novel microbial isolates and genes that may be beneficial as biofertilizers, biopesticides, and biostimulants in an era of climate change.

Plant growth-promoting rhizobacteria (PGPR) strains are able to produce IAA, solubilize phosphate, induce ACC deaminase, and chelate iron by producing siderophore. Therefore, their application is an effective means of alleviating stress in planted crops (Etesami and Jeong, 2018). The PGPR strains achieve improvement in the growth and tolerance of planted crops through the accumulation of compatible solutes like proline or glycine betaine, by enhancing the secretion of bioactive metabolites, as well as through inducing the expression of plant growth beneficial genes.

Recently, the Metabolomics profiling of *Sorghum bicolor* that was primed with PGPR isolates (*Bacillus* and *Pseudomonas*) and exposed to drought stress, induced systemic tolerance in the plants (Carlson et al., 2020).

Also, proteomic analyses of *Medicago truncatula* that was inoculated with *Sinorhizobium* sp. and exposed to drought stress, led to the upregulation of JA pathway and downregulation of ethylene biosynthesis which are vital for improved drought tolerance (Staudinger et al., 2016).

In addition, the inoculation of *Trichoderma* and *Pseudomonas* in rice plants that was subjected to drought stress induced the overexpression of antioxidative enzymes and the phenylpropanoid biosynthesis pathway, as well as other key drought responsive genes (Singh et al., 2020).

PGPR remains a promising option for improving crop drought resistance, as reveal in a transcriptomics study by Morcillo et al. (2021) applying the bioinoculant B. megaterium TG1-E1 on different tomato cultivars under drought conditions. The findings reveal several key mediators of TG1-E1-induced transcriptional regulation in tomato plants, including transcription factors, stress signaling components and regulators, and putative regulators of cell wall organization. Also, analysis of the metabolites indicated the presence of important compounds that include ethanolamine, amino acid, sugars, and pinitol, which aided in TG1-E1-triggered plant drought resistance.

By using high-throughput RNA-sequencing techniques (Thomas et al., 2019), characterized differentially expressed genes (DEGs) in rice roots upon inoculation with *A. brasilense*. The findings reveal plant growth promotion impacts, pathways and genes that are involved in the plant-microbe interactions.

Furthermore, in a study by Zhang et al. (2020) using culture independent 16S rRNA gene amplicon sequencing and culture-dependent functional analyses of *Alhagi sparsifolia* rhizosphere and root endospheric microbiome, identify key endophytic bacterial taxa and their genes facilitating drought resistance in wheat. Through comparative genomics analysis, a drought resistance-promoting strain was characterized, as well as the mechanisms deployed in colonization and enhancement of drought resistance in wheat was elucidated.

### Deployment of Climate-Smart Agricultural Practices for Improving Productivity

One of the major challenges faced by humanity over the ages has been the task of tackling in a sustainable way environmental degradation and the consequences of climate change which are more pronounced in the case of natural ecosystems (Sarkar et al., 2020). The effects of climate change are more pronounced in agro-ecosystems because the sum total of all agricultural activities takes place on them and that is why they are the most vulnerable of all of the natural ecosystems (Dubey et al., 2020).

**Figure 2:**
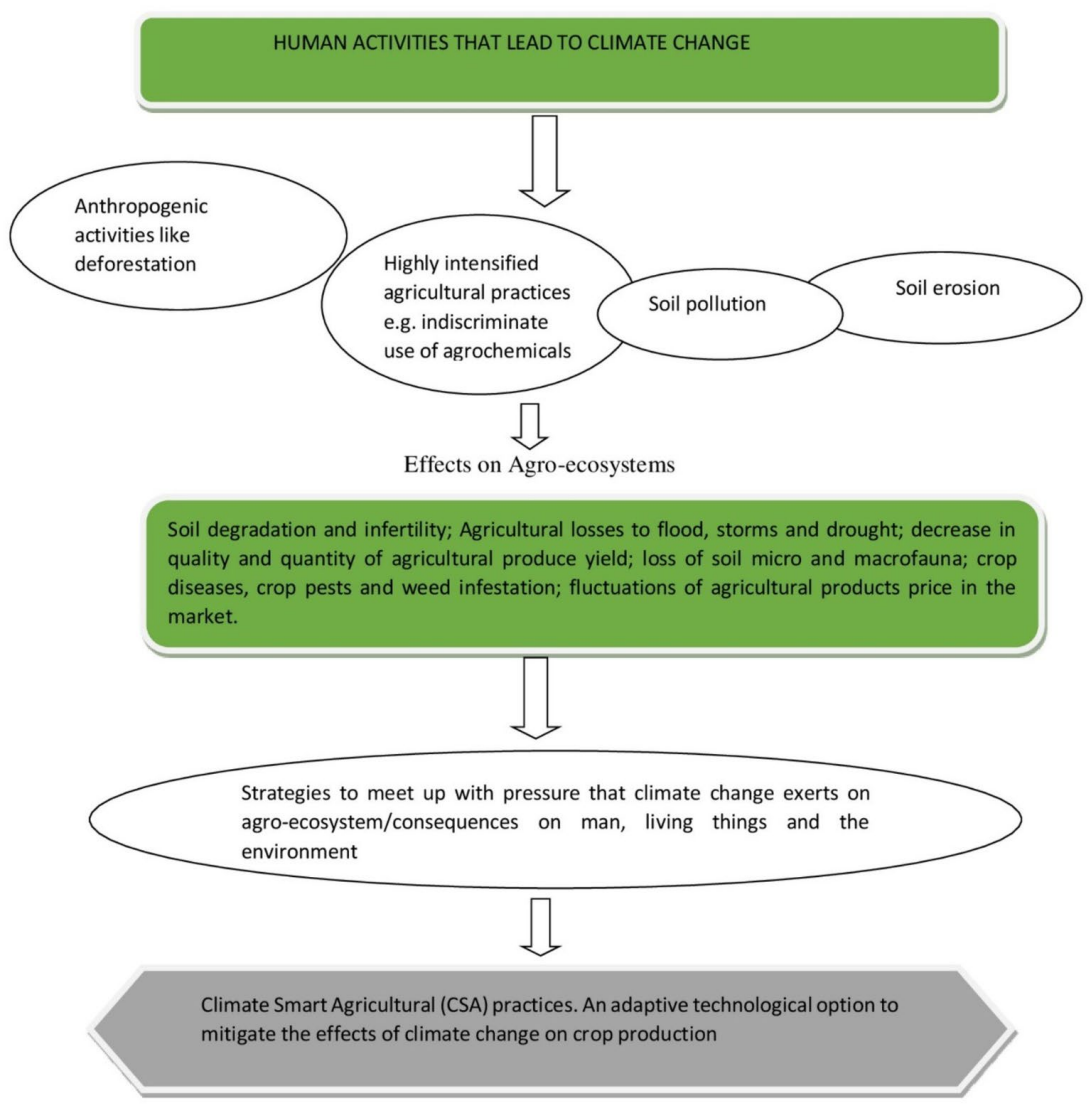
Schematic highlighting of the different pressures exerted by climatic change and CSA as a mitigating practice to improve agricultural production.

The deployment of ecologically and environmentally unfriendly practices such as the excessive intensification of agricultural practices on the land, the indiscriminate use of agrochemicals, such as pesticides, herbicides and fertilizers, as well as the consequences of anthropogenic activities, such as like urbanization, deforestation, industrialization, and the burning of fossil fuels, collectively result in greenhouse gas (GHG) emissions and the ultimate disruption of the agro-ecological balance (Lawrence and Vandecar, 2015; Dubey et al., 2016). To meet up to the challenges posed by the high consumption levels of a rapidly growing population has proved to be a huge task. This is especially true for the developing world where, under the changing climatic conditions, there is a need to adopt strategies and practices that are socially, economically, and ecologically acceptable in the management of our natural resources (Abhilash et al., 2016; Sarkar et al., 2017). Climate-smart agriculture presents various innovative practices that can be adopted to meet the global food demand while concomitantly mitigating the effects of unfavorable climatic conditions on the production of climatically vulnerable crops. CSA is based on existing knowledge, technologies, and sustainable agriculture (FAO, 2015) and presents an integrated approach to managing cropland, livestock, forests, and fisheries in order to achieve food security, reduced greenhouse gas emissions and to contribute to other development goals in the face of climatic changes (Palombi and Sessa, 2013; Figure 2).

According to Kumar et al. (2019), some of the CSA practices and technologies are able to mitigate the effects of climate change on the agro-ecosystem, to boost agricultural production and to reduce the effects of GHGs. They include the use of quality seeds and the planting of well-adapted crops, effective biodiversity management, and integrated pest management systems, efficient water management, sustainable land and soil management to ensure increased crop production, and sustainable and efficient mechanization.

Other CSA mitigation practices include low-input sustainable agriculture (LISA) practices, which focus on safe farming and that incorporate local knowledge of the farming system, and in so doing, produce abundant, nutritious, profitable food products without causing negative effects to both the natural agro-ecosystem and human health (Najafabadi et al., 2012). According to Sarkar et al. (2015) indigenous technical knowledge (ITR) concerns the knowledge that local people have gathered through their interactions with nature and that has allowed them to adopt mitigating measures to counter the effects of climate change and thus to boost their crop production.

Also, simulation model studies are vital tools that can be used to conduct studies of different agro-ecological regions in order to implement sustainable agricultural measures, to achieve effective and maximum production levels (Sarkar et al., 2020). Organic farming also goes a long way to reducing the effect of GHG emissions (Rakshit et al., 2010).

Importantly, (Cammarano et al., 2020) used the Agricultural Model Intercomparison and Improvement Project (AgMIP) as a tool that, in the face of the prevailing drought problem in the northeastern area of Free-state, South Africa, incorporated data about climate change, crops and the economy to provides and implement adaptation strategies to improve and increase the production of maize in this region. Likewise, (Ishikawa et al., 2020) used the farmers’ participatory varietal selection (FPVS) method to collect information from local farmers in the southern regions of Burkina Faso, in West Africa. They used the collected data to gather information on how to breed and select newly improved drought-resistant cowpea seeds for maximum production, which would prove to be economically and socially beneficial.

### Prospects of Advanced Multi-omics Biotechnological Techniques for Improving Cowpea Productivity

In this modern era, where there is a notion of smart biotechnological techniques that can turn around the immense challenges of optimizing agricultural system outputs productivity, the multi-omics biotechnological tools are usually the game-changer. These multifaceted biotechnological techniques encompassing genomics, transcriptomics, proteomics, and metabolomics offer great prospects for improving crop protection, crop yields/productivity, and for ensuring nutritional food sources that are safe and secured for human consumption.

Through the application of the techniques of genomics, transcriptomics, proteomics and metabolomics, plant breeding has improved, and biotic and abiotic stress-resistant and resilient crop cultivars have been developed, thus leading to the production of better-quality crops.

Multi-omics biotechnological tools encompass a knowledge of analytical chemistry, computational biology, and bioinformatics analysis, as well as other thematic areas of biology, to facilitate a systematic approach to research studies, which would then lead to crop production and productivity enhancement.

Metabolites, proteins, and genes are specific components that are targeted and researched to improve crop cultivars and to better understand their growth characteristics.

These smart biotechnological techniques are advanced, concise, precise, and valuable tools that can be specifically targeted for improving crops. In fact, they are vital tools for sparking the latest green revolution in agricultural productivity. They can be used to introduce genes, proteins, or metabolites of interest with good traits to improve and intensify the productivity of planted crops. Thus, fewer agro-resource inputs would then be necessary in agricultural systems to attain better agro-product outputs.

Multi-omics biotechnological tools can be deployed to reveal key information on (plants and microbes). Furthermore, these tools could be applied to orchestrate metabolic and physiological changes, and also in genetic engineering for crop improvement (Chassy, 2010). Multi-omics techniques can also be used in breeding transgenic crops with specific key agronomic traits (Ahmad et al., 2012).

The multi-omics biotechnological tools, namely genomics, transcriptomics, proteomics, and metabolomics, are inter-woven techniques, that are closely linked and that can be applied to overcome the daunting challenges of feeding the burgeoning global population in this era of climatic vulnerabilities. They can also be deployed to consolidate the foremost producer status of the African continent in that they are able to enhance cowpea productivity and production.

#### Genomics

Genomics is the foremost pioneer omics that is presented as an advanced biotechnological technique and that uses genes and the genome transformation of plants and microbes for molecular breeding in order to establish improved crop cultivars. Genomics techniques are fast and precise, and can be selectively used to highlight the functional genes of desired traits for the improvement of a plant. Specifically, genomics techniques can be applied in the modification of genes in that they add genes to a plant, or by using RNAi, they knock down genes from a plant, in so doing, accomplish phenotypic traits of interest faster than the conventional plant-breeding method does. In the quest to enhance cowpea productivity, genomics-based smart biotechnology techniques have been deployed to breed improved cowpea cultivars. In such cases, the focus is on looking at the whole genome in terms of genotypic diversity and fingerprinting for cultivar improvement traits (Xu et al., 2017; Wu et al., 2018; Seo et al., 2020).

Molecular-based approach has been deployed towards improving cowpea cultivars using molecular markers and genomic-breeding techniques. An authenticated cowpea genetic resource is the foundation for efficient breeding and conservation. Genotypic diversity assessment is done by using both phenotypic and molecular traits characterization.

Research efforts at genetic breeding of cowpea cultivars using these DNA markers have been investigated by Kolade et al. (2016); Chen et al. (2017a).

SNPs are the preferred markers in genotypic assessment studies due to their wide distribution in the genome and they are highly efficient (Nkhoma et al., 2020).

Following advancement in plants genome resources, molecular markers are now widely deployed in genetic variability assessment, molecular breeding, and DNA fingerprinting (Su et al., 2018).

Among the genomic-breeding research effort, the Illumina Cowpea iSelect Consortium Array (Muñoz-Amatriaín et al., 2017) was an important landmark. This great research effort led to the development of a minicore (referred to as the “UCR Minicore”) which composed of 368 domesticated cowpeas selected from a larger set of _5000 accessions comprising the UC Riverside cowpea collection.

This array contained 51,128 SNPs derived from whole genome sequences (WGS) of 37 diverse cowpea accessions. Single nucleotide polymorphism (SNPs) is distributed uniformly in cowpea genome and indicates variation in genes of cowpea. Thus, they provide an ideal resource for cowpea molecular breeding and new variety protection. SNPs are vital genomics techniques for assessment of key traits in cowpea like constructing genomic linkage map, for QTL, for the detection as well as assessing germplasm genetic diversity (Paudel et al., 2021).

Also, the majority of the international institute of tropical agriculture (IITA) minicore collection (298 accessions) was genotyped using genotypic base sequencing (GBS) with 2,276 SNPs, this identified three major subpopulations (Fatokun et al., 2018), but showed dispersion of West and Central African accessions across the three subpopulations.

Another giant stride in the progress of cowpea genomics study was achieved by using next generation sequencing advancement (Lonardi et al., 2019) to authenticate the whole genome of an improved cowpea genotypes, thus providing a key resource that is crucial to deciphering the morpho-physiological response of cowpeas.

Building on this developments and report of full SNP data release for the UCR Minicore, numerous follow up studies has been investigated for more focus cowpea research, that include studies on pattern of seed coat (Herniter et al., 2019), color of seed coat (Herniter et al., 2018), size of seeds (Lo et al., 2019), resistance to bruchid infestation (Miesho et al., 2019), plant herbivore resistance (Steinbrenner et al., 2020) and pod shattering (Lo et al., 2021).

With better comprehension of genomic basis of variation, genome-wide association studies (GWAS) studies have been highlighted in cowpea for pod length (Xu et al., 2017), root architecture (Burridge et al., 2017), cowpea plant improvement traits, as well as the flowering period (Muñoz-Amatriaín et al., 2021). All these findings are appreciated because cowpea genetic diversity assessment is necessary for strengthening breeding programs in order to develop high yielding dual-purpose cultivars with good grain and fodder yields.

#### Transcriptomics

Transcriptomics is a vital biotechnological technique that makes for a comprehensive understanding of genomics functionality (Valdés et al., 2013). Transcriptomics regulates the expression of genes in the context of biotic and abiotic stresses. Transcriptomics is a dynamic technique that expresses genes at any given time and under different circumstances.

With the advancement of functional genomics, the identification of novel genes having vital functions in plant growth/development and adaptation to stressful conditions have been characterized for crop cultivars (Zhang et al., 2017). Also, RNA expression profiling is important in understanding plant functionality.

Transcriptomics as a part of multi-omics biotechnological techniques have led to the detection of novel genes useful in response to both biotic and abiotic stresses in plants.

Transcriptomics approaches utilizes high-throughput sequencing platforms to generate enormous useful transcript data through techniques such as RNA sequencing, microarray and serial analysis of gene expression (SAGE) to elucidate non-coding and coding RNAs expression profiles to plant biotic and abiotic stresses (Cramer et al., 2011; Santos et al., 2018).

Several factors like the duration and extent of stress conditions, determines the adaptability and tolerance of a plant to stresses. However, experimental design, handling of tissue samples, isolation of RNA and stability of RNA also play major role in any transcriptomic analysis (Gokce et al., 2020).

The characterization of different parts of the cowpea plant through transcriptomics has been carried out in studies that express the diverse genes essential for cowpea growth and development. The stress-resilient genes have also been characterized and their role in the overall improvement of cowpea has also been highlighted (Yao et al., 2016; Chen et al., 2017b; Amorim et al., 2018; Spriggs et al., 2018).

#### Proteomics

Proteins are a vital constituent of plants. Large quantities of protein are responsible for the key functional roles that plants perform. As a smart biotechnological technique, proteomics entails the expression of functional characteristics, structural features, and the translation/manifestation of beneficial traits in plants. Another important attribute of the proteomics technique is that it can be used to better elucidate a pesticide’s mode of action, its mechanisms, and its biodegradation. The outputs/benefits that can be derived by applying proteomics include the authenticity of the food product, the assurance of food security that it represents and the sustainability of energy that the food product offers to consumer, as well as the maintenance of an environmental balance (Agrawal et al., 2012; Landim et al., 2017).

Proteomics as a key branch of “omics” technology aims at investigating protein’s structure, function, as well as their interactions with other proteins and other components, including the modifications arising from these interactions through the use of analytical techniques.

Proteomics approach involves analysis and the elucidation of functional expression of proteins in order to understand biological processes (Iwamoto and Shimada, 2018; Chen and Weckwerth, 2020).

Proteins are vital components of all biological process. To fully comprehend the response of plants to biotic and abiotic stresses, proteomics studies must be assessed, along with other multi-omics technology (Gokce et al., 2020). Changes in gene expression influences appropriate response in protein composition/abundance and affect cellular functions.

Proteomics studies are assessed using spectroscopic method usually by mass spectroscopy (MS)-based technology. This is done by MALDI-TOF MS or with liquid chromatography mass spectrometry (LC–MS) techniques. Proteomics studies have led to the characterization of different stress response proteins in planted crops under stress conditions (Rathi et al., 2016; Kosová et al., 2018; Matamoros and Becana, 2021).

#### Metabolomics

Metabolomics is an advanced and powerful smart biotechnique that identifies functionally active metabolites, their roles, and the diverse biochemical processes that the metabolites play in plant genotypes and phenotypic expressions (Führs et al., 2009; Aliferis and Chrysayi-Tokousbalides, 2011). Metabolomics tools can be deployed in identifying and monitoring physiological responses in plants and the metabolic pathways or linkages arising from the biotic and abiotic stress exerted upon plants. In fact, these tools are able to enhance crop development and improve plant health (Dixon et al., 2006; Goufo et al., 2017).

In a study on drought response of three cowpea landraces using leaf physiological and metabolites profiling assessment, (Gomes et al., 2020), used gas chromatography time of flight mass spectrometry (GC-TOF-MS) and reported that cowpea landrace A116 genotype drought response was best with the accumulation of 14 bioactive metabolites that included proline, valine, and rhamnose and raffinose, isoleucine, fucose, urea, alanine, sucrose, and putrescine.

Also, in a study on metabolites (polyphenols and carotenoids) in *V. unguiculata* sprouts by Yeo et al. (2018), investigated using high-performance liquid chromatography (HPLC), electrospray ionization-mass spectrometry (ESI-MS), gas chromatography–mass spectrometry (GC–MS), and gas chromatography, 39 hydrophilic compounds were identified and quantitated. Thus, the study provides a new approach for enhancing the carotenoid and phenylpropanoid production of *V. unguiculata*.

Metabolomics as a powerful omics-based approach can be applied as a tool to explore different aspects in plant breeding, the regulatory mechanisms related to plant growth and development (including those related to crop productivity and performance), adaptation to biotic and abiotic stresses, nutritional improvement, and selection of cultivars for agriculture. Metabolomes are simply metabolites (both secondary and primary) having low molecular weight (usually <1,500Da), including their precursors and intermediates of the corresponding biosynthetic pathways. Such compounds are considered the end products of gene expression and protein activity, modulating processes between the genome and environment and indicating the functional status of the organism. Moreover, they are an indispensable part of the plant metabolism, influencing all biological processes, such as plant biomass and architecture, and those involved in plant defense or adaptation to biotic and abiotic stresses (Sharma et al., 2021).

In a comprehensive study on cowpea osmoregulation response under drought stress, (Goufo et al., 2017) investigated and provided a detailed metabolic profile of a broad range of primary and secondary metabolites in cowpea, including elemental solutes using (leaves and roots). Their findings revealed that the mechanisms deploy in modifying cowpea metabolism response to water deficit is through interplay between the shikimate and arginine/proline pathways, leading to three drought-responsive metabolites, namely galactinol, proline, and quercetin 3-O-6''-malonylglycoside.

In a study aimed at identifying metabolic responses and key factors associated with Mn tolerance using Mn-tolerant and Mn-sensitive genotypic cultivars; (Führs et al., 2012) reported that manganese tolerance is a consequence of genotypic/constitutive higher concentrations of metabolites detoxifying manganese and reactive oxygen species.

### Agricultural Conservation Practices for Crop Productivity Enhancement

Agricultural conservation practices are simple and cost-effective techniques for achieving sustainable productivity enhancement in planted crops. This technique is based on the use of a limited number of natural resources as inputs. Crop rotation, mixed farming methods, intercropping, the manual tillage of the soil, and the use of crop residues to reduce soil moisture loss through mulching are some of the methods employed. These simple, cost-effective techniques, using a limited number of resources as inputs, ultimately lead to crop productivity enhancement.

However, in order to effectively enhance crop productivity, it is necessary to find effective ways of adapting to climate change and the vulnerability it imposes on crops and farmers. The objective should always be to mitigate the adverse impacts of climate change on the environment (Lipper et al., 2014).

Conservation agriculture improves the quality of the soil – biologically, physically, and chemically, and thus ultimately makes an impact on the crop production outputs, with both positive and sustainable effects (Basavanneppa et al., 2017). In addition to improving crop yields and achieving sustainability, conservation agriculture also augments microbial diversity and enhances microbial functionality (Yadav et al., 2017).

Conservation agriculture is increasingly being promoted as an adaptive climate-smart agricultural technique that can minimize the adverse effects of synthetic agrochemical usage in agricultural systems that generally lead to poor and depleted soil fertility (Pretty and Bharucha, 2014).

As an agroecological system tool, conservation agriculture can lead to enhanced crop productivity, the diminished use of agro-resource inputs, environmental sustainability, and advance the income generation potential of farmers (Prasai et al., 2018; Pariyar et al., 2019).

Conservation agriculture helps in enhancing soil fertility and in reducing the cost of the associated inputs. The application of conservation practices improves soil water conservation and soil moisture, minimizes runoff, reduces moisture losses through evaporation, boosts the biological properties of the soil, and enhances crop productivity (Hossain et al., 2015).

The beneficial effects of conservation agriculture on crop productivity can be classified into three main categories:

1. Conservation agriculture provides agronomic growth benefits and enhances soil health.
2. The sustainability of the environment and the soil and the sociological benefits of the agricultural production system are enshrined.
3. Conservation agriculture can lead to enhanced economic benefits and also improve efficiency in the agricultural sphere.

In a nutshell, conservation agricultural practices enhance the quality of planted crops, improve the fertility of the soil, and ultimately provide both socioeconomic and environmental benefits in a sustainable manner (Bell et al., 2019; Calcante and Oberti, 2019).

The applications of bio-based, renewable, agro-ecologically balanced, and advanced smart biotechnological techniques in achieving improvements in the productivity of cowpea and a few selected crops of economic importance are presented in Table 3 as effective sustainable alternatives for crop improvement.

**Table 3:** Sustainable deployment of bioinoculants and smart biotechnological techniques for the productivity enhancement of cowpea and some selected food crops.

Beneficial microbial inoculants	Crop of interest	Bioactive roles of inoculants	References
*Co-inoculation of Bradyrhizobia strains*	*Vigna unguiculata*	Growth improvement of cowpea	Do Nascimento et al., 2021
*Mutant of Glomus sp. and Trichoderma harzianum (AMF60+TH)*	*Vigna unguiculata*	Used for growth promotion and biocontrol of powdery mildew disease of cowpea	Omomowo et al., 2018
*Mutant strains of Glomus versiforme and Trichoderma harzianum*	*Vigna unguiculata*	Used for growth promotion and biocontrol of Cercospora leaf spot disease of cowpea	Omomowo et al., 2020
Genome-wide association studies (GWAS)	*Vigna unguiculata*	Enhancement of drought tolerance of cowpea	Ravelombola et al., 2021
Chitin-binding protein studies (CBV)	*Vigna unguiculata*	Toxic influence and reduction in larval mass and length of *Callosobruchus maculatus* (Cowpea weevil)	Ferreira et al., 2021
Genome-wide association studies (GWAS), meta-analysis and Sequence homology combination	*Vigna unguiculata*	Identification of candidate genes for cowpea seed size enhancement	Lo et al., 2019
Synergistic effects of co-inoculation with different AMF isolates and *Sinorhizobium meliloti*	*Vigna unguiculata*	Enhancement of above ground biomass production and nitrogen content	Kavadia et al., 2021
QTL mapping using recombinant inbred line (RIL) and transcriptome analysis	*Vigna unguiculata*	Identification of candidate genes for root-knot nematode resistance (Rk) in cowpea	Santos et al., 2018
SSR typing for diversity assessment and nitrogen fixation potentials	*Vigna unguiculata*	Identification of SSR marker for nitrogen fixation and other symbiosis-related traits	Mohammed et al., 2020
Synergistic influence of *Trichoderma* and *Bradyrhizobia* on cowpea growth improvement	*Vigna unguiculata*	Enhancement of cowpea growth biomass and photosynthetic pigments	Mendes et al., 2020
Proteomic approaches using miRNAs and Argonaute genes in response to CPSMV stress	*Vigna unguiculata*	Detection of miRNAs and genes that elicits a response to CPSMV	Martins et al., 2020
Transgenic cowpea plant response to *Maruca vitrata* legume pod borer	*Vigna unguiculata*	Improvement in the prevention of damage caused by pod borer due to genetically engineered cowpea	Kumar et al., 2021a
Deployment of Entomopathogenic fungi together with intercropping in managing *Aphis craccivora* infestation of cowpea	*Vigna unguiculata*	Reduction in the damage caused by an aphid infestation of cowpea	Mweke et al., 2020
Deployment of conservation agricultural practices of no-tillage and planting of cover crops	*Vigna unguiculata*	Improvement in soil carbon and nitrogen nutrient concentration, as well as good adaptation to water stress	Guzzetti et al., 2020
Deployment of yeast isolates in controlling *Rhizoctonia solani* infestation in cowpea	*Vigna unguiculata*	Effective in the biocontrol of damping-off and stem rot of cowpea plants caused by *R. solani*	De Tenório et al., 2019
Deploying encapsulated *Pseudomonas libanensis* in alleviating cowpea drought stress	*Vigna unguiculata*	Encapsulation of the beneficial microbe highlighted its positive impact on managing drought stress in cowpea	Souza-Alonso et al., 2021
Application of embryonic axis explants for efficient regeneration, transformation, and genome editing of cowpea	*Vigna unguiculata*	CRISPR/Cas was used successfully to develop transgenic cowpea plantlet	Che et al., 2021
Application of *Bacillus subtilis* Dcl1in cowpea plant as growth enhancer, biocontrol, and abiotic stress abatement agent	*Vigna unguiculata*	Improvement in cowpea growth, biotic and abiotic stress effectors	Jayakumar et al., 2021
Deployment of MgO nanoparticles in enhancing cowpea growth and controlling nematode infestation	*Vigna unguiculata*	Improvement in cowpea growth and control of root-knot nematode infestation	Tauseef et al., 2021
Deploying *Bacillus sp.* Fcl1as pesticide toxicity alleviating and growth-promoting impact on a cowpea plant	*Vigna unguiculata*	Improvement in cowpea growth and also toxicity alleviating effects of pesticide	Juby et al., 2021
Application of *Bacillus cereus* NDRMN001 and *Kosakonia sp.* MGR1 to improve cowpea growth and remediate heavy metal toxicity	*Vigna unguiculata*	Enhancement in the growth characteristics of cowpea plant and also the remediation of heavy metal toxicity	Narayanan et al., 2021
Inoculation of *Bradyrhizobium* and salicylic acid effects in mitigating water stress deficit in cowpea plant	*Vigna unguiculata*	Effective in the improvement of cowpea growth, proline content, superoxide dismutase, and ascorbate peroxidase	De Andrade et al., 2021
Inoculation using *Bradyrhizobium* BR3267 with phosphorus and potassium fertilizer improves cowpea growth	*Vigna unguiculata*	The combined inoculant treatment was effective in increasing cowpea yield and growth parameters	Emmanuel et al., 2021
Interactive influence of *Bacillus subtilis* that were co-inoculated with mine water on the physiological and nutritional growth enhancement of cowpea	*Vigna unguiculata*	*Bacillus subtilis* co-inoculated with mine water, sequester heavy metals, and improve nutritional content and growth of cowpea	Nevhulaudzi et al., 2020a
Influence of inoculation using dark septate endophytic fungi on cowpea productivity under salinity stress	*Vigna unguiculata*	Improvement in nutritional content and photosynthetic rate of cowpea plant	Farias et al., 2020
Application of indigenous mycorrhizal and nano-Ti0_2_ in reducing cowpea oxidative stress and Cd uptake	*Vigna unguiculata*	There was a reduction in both the Cd metal uptake and oxidative stress of cowpea due to co-inoculation treatment	Ogunkunle et al., 2020
Response of field-grown cowpea to inoculation with *Bradyrhizobium*	*Vigna unguiculata*	Improvement in agronomic growth parameters of cowpea plant due to bioinoculant treatment	Ayalew et al., 2021
Seed inoculant treatments using rhizobacteria and mycorrhizal improve the growth and nutrition of cowpea under water stress	*Vigna unguiculata*	Improvement in growth and nutritional content of cowpea due to mycorrhizal and rhizobacteria application *via* seed coating	Rocha et al., 2020
Inoculation with Rhizobia strains and AMF species	*Glycine max*	Yield and nutrient improvement of soybean	Igiehon et al., 2021
Inoculation with *Rhizobium* and Mycorrhizal Fungi species	*Glycine max*	Yield improvement of soybean under drought stress	Igiehon and Babalola, 2021
Inoculation with *Trichoderma* Isolates	*Glycine max*	Biocontrol of destructive nematode of soybean	De Oliveira et al., 2021
*Bacillus sp*. PS2 and PS10	*Zea mays*	Plant growth and yield enhancement of Maize	Chaudhary et al., 2021
Mixed inoculation of *Bacillus cereus* BI-8 and *Bacillus subtilis* BI-10	*Zea mays*	Plant growth and nutrient yield enhancement of Maize	Fouda et al., 2021
*Azotobacter chroococcum*	*Zea mays*	Soil health improvement and nutrient yield enhancement of Maize	Song et al., 2021
Application of different Microbial inoculants	*Wheat*	Improvement in wheat growth and soil microbiome diversity	Chen et al., 2021
Inoculation with endophytic fungi *Nectria haematococca*	*Green gram*	Growth and nutritional improvement of Green gram	Muthukumar and Sulaiman, 2021
Inoculation with Potassium solubilizing B*acillus cereus*	*Potato*	Growth and yield improvement of potato	Ali et al., 2021
Application of different Arbuscular Mycorrhizal fungi	*Cicer arietinum L*	Improving Arsenic metalloid tolerant and yield of chickpea	Garg and Cheema, 2021
*Bacillus spp*	*Pearl Millet*	Used as a biocontrol agent for fungal pathogens affecting Pearl millet	Kushwaha et al., 2020
*Bacillus subtilis*	*Oryza sativa*	Biocontrol agent for control of fungal disease of rice	Kumar et al., 2020
*Bacillus pumilus* strain JPVS 11	*Oryza sativa*	Improving growth/yield and salinity tolerance in rice	Kumar et al., 2021b
Inoculation with *Piriformospora indica*	*Oryza sativa*	Improving yield and arsenic tolerance in rice	Ghorbani et al., 2021
Single and co-inoculation with mycorrhiza	*Phaseolus vulgaris*	Improving yield and nutrition of snap bean	Beltayef et al., 2021
Inoculation with single and co-inoculation with AMF and PSB	*Zea mays*	Improvement in productivity of maize	Pacheco et al., 2021
Inoculation with *Funneliformis mosseae*	*Triticum aestivum L.*	Improving wheat productivity and enhancing soil health	Duan et al., 2021
Single and co-inoculation with *Piriformospora indica* and *Pseudomonas putida*	*Triticum aestivum L*	Enhancement in growth and nutritional status of wheat	Abadi et al., 2021

## Mechanistic Physiological Processes/Actions Involved in the Deployment of Smart Biotechnological Techniques to Improve Cowpea Productivity

**Figure 3:**
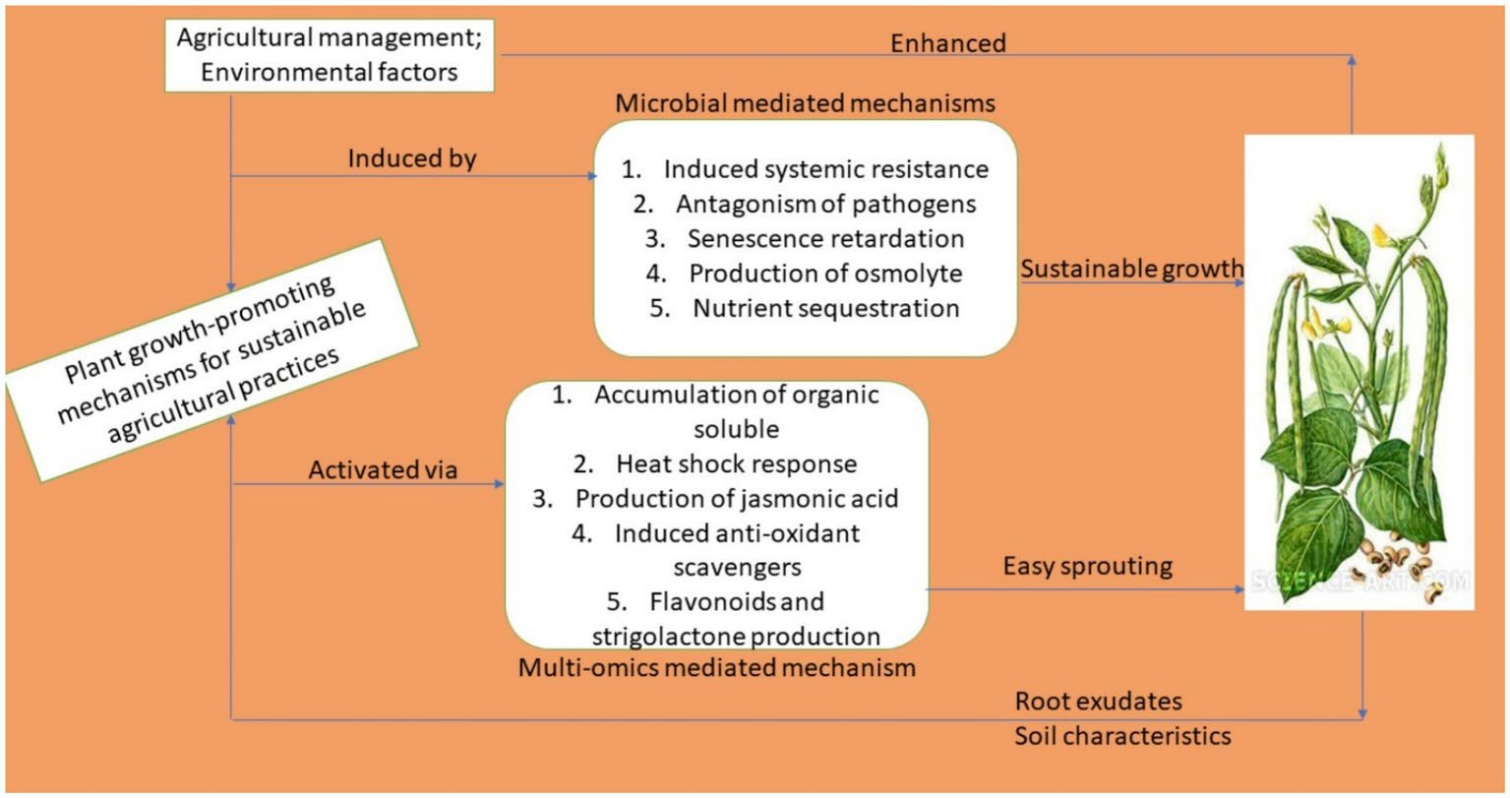
Mechanisms of action of smart biotechnological techniques deployed in cowpea productivity enhancement.

Owing to the application of smart biotechnological techniques, diverse physiological and metabolic modes of activities are involved in improving the productivity outputs of cowpea. This can be achieved through direct and indirect modes of action such as those involved in, among others, directly supplying nutrients to plants, suppressing phytopathogens through the production of plant growth effectors, regulating the hormonal balance of plants, triggering various immune responses, and through the secretion of vital proteins (Santos Villalobos et al., 2018; Villarreal-Delgado et al., 2018). An overview is presented in Figure 3.

## Concluding Remarks and Future Perspectives

**Figure 4:**
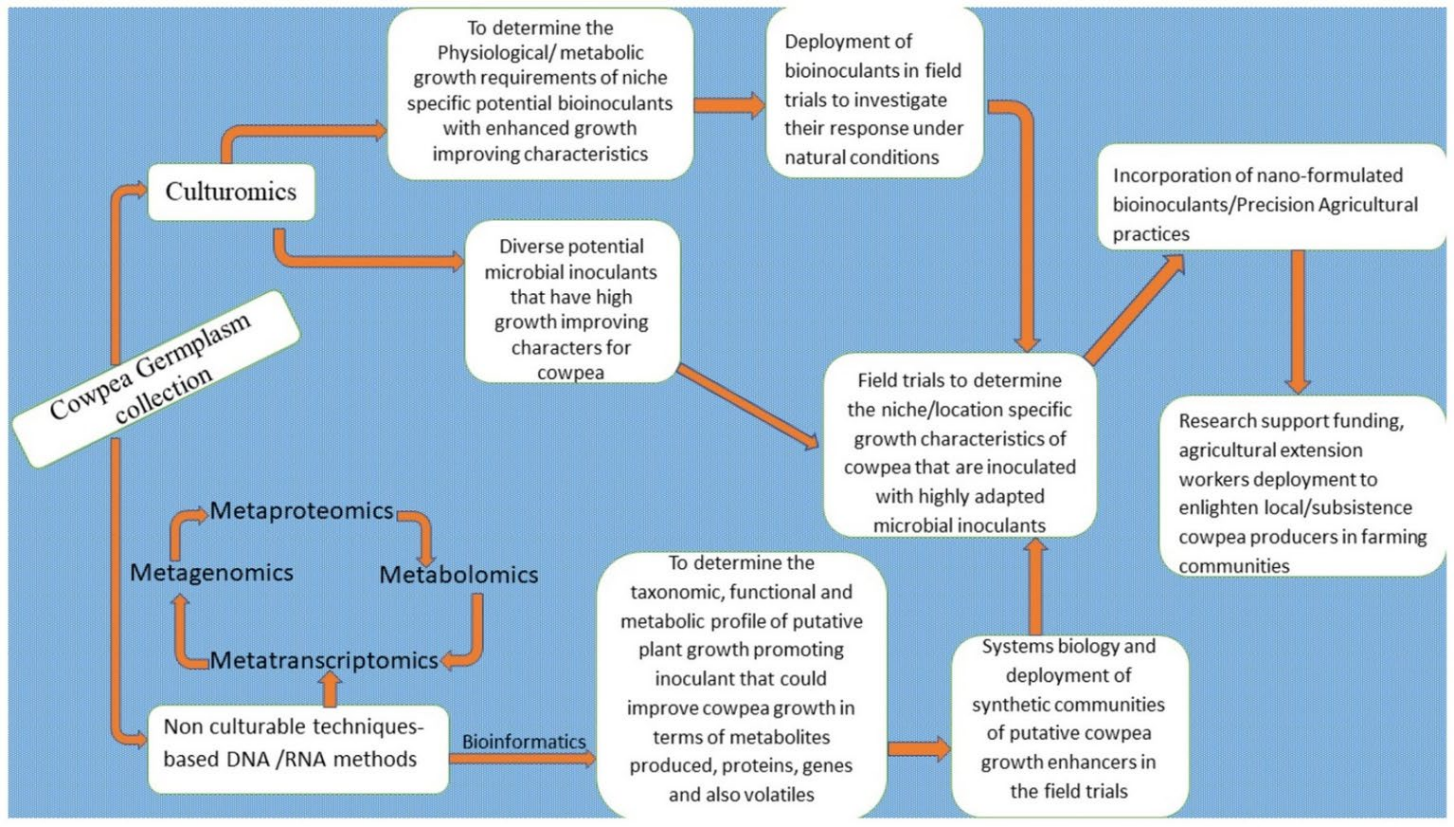
Holistic approach and futuristic perspectives for improving cowpea productivity enhancement in Africa and consolidating the continent’s foremost producer status.

Yes! Daunting and herculean are the constraints that almost all African nations face in terms of improving their crop yields and productivity in the light of the current global challenges. These are aggravated by the global pandemic, climatic change, and a burgeoning population growth rate. However, a concerted effort directed at achieving the sustainable development goals of reducing poverty and eliminating hunger and malnutrition is what is called for. The first priority is to strive for an improvement in the agricultural system. The use of agro-ecologically balanced improvement techniques remains the surest way to achieve this. The constraints of low yields and the limited productivity of the cowpea, a valuable indigenous African legume at the forefront on the continent in terms of its potential as a food product, were highlighted in this review. The prospect of circumventing and overcoming these constraints is in fact a very real possibility. An essential requirement would be the use of viable tools. These would include the deployment of sustainable, ecosystem-friendly smart biotechnological tools: the application of bioinoculants, climate-smart agricultural practices, agricultural conservation techniques, as well as advanced multi-omics biotechnological tools for the improvement in cowpea yields and productivity enhancement. However, there are research gaps that still need to be worked upon to ensure success. Several collaborative efforts should be directed at building the capacity of plant breeders, agronomists, biotechnologists, and other allied stakeholders in the agri-food value chain in Africa to embrace these sustainable biotechnological techniques. Further research efforts should be directed at attaining specific functional traits in cowpea plants, in order to develop locality adaptive and climate-specific traits – the latter in response to climatic vulnerabilities and other external stressors – all for the benefit of the planted cowpea crop. Furthermore, efforts should also be directed at exploring an integrative and holistic approach to systematic biology that would combine systemic knowledge in the field of multi-omics biotechniques, genetic engineering tools, precision agricultural practices, techniques in genome editing technology (CRISPR/Cas), synthetic biology, bio-computational technology, as well as the emerging field of agro-nanobiotechnology for the improvement of the cowpea crop. The use of a synthetic microbial consortium, (SYNCOMs) should be deployed to the field to vigorously phenotype cowpea cultivars that are trait-specific and can be grown as a crop adapted to a niche environment, and favored by most cowpea producing marginal communities in Africa (Figure 4).

There is, however, a need to integrate socioeconomic policy into this sound biotechnological know-how system in order, to reach a balance, as well as a guaranteed and steady flow of the necessary financial support for the associated research efforts. Attention should also be directed to developing a policy of backward integration to achieve positive and sustainable results in the context of improving and enhancing the productivity and yields of cowpea, a key leguminous crop that is considered to be of great importance in Africa.

## Author Contributions

OOB conceptualized the topic. OIO conducted the literature search and undertook the drafting and writing of the manuscript, while the final editing was carried out by OOB. The authors made substantial intellectual contributions to the work and agree to be accountable for the content of the work.

## Funding

This work was supported by a grant from the National Research Foundation of South Africa (grant ref: UID123634 and UID132595 OOB).

## Conflict of Interest

The authors declare that the research was conducted in the absence of any commercial or financial relationships that could be construed as a potential conflict of interest.

## Publisher’s Note

All claims expressed in this article are solely those of the authors and do not necessarily represent those of their affiliated organizations, or those of the publisher, the editors and the reviewers. Any product that may be evaluated in this article, or claim that may be made by its manufacturer, is not guaranteed or endorsed by the publisher.
