# Corrigendum: Constraints and prospects of improving cowpea productivity to ensure food, nutritional security and environmental sustainability

**DOI:** 10.3389/fpls.2022.1042678

**Published:** 2022-10-06

**Authors:** Olawale Israel Omomowo, Olubukola Oluranti Babalola

**Affiliations:** Food Security and Safety Niche Area, Faculty of Natural and Agricultural Sciences, North-West University, Mmabatho, South Africa

**Keywords:** cowpea productivity enhancement, indigenous legume, *Vigna unguiculata*, nutritious human food, the largest producer status, smart biotechnological approaches, protein-rich fodder-for livestock

## Error in figure table

In the published article, there was an error in **Figure 1** as published. There was an error during the stage of joining the pictures A to G together.

**Figure 1:**
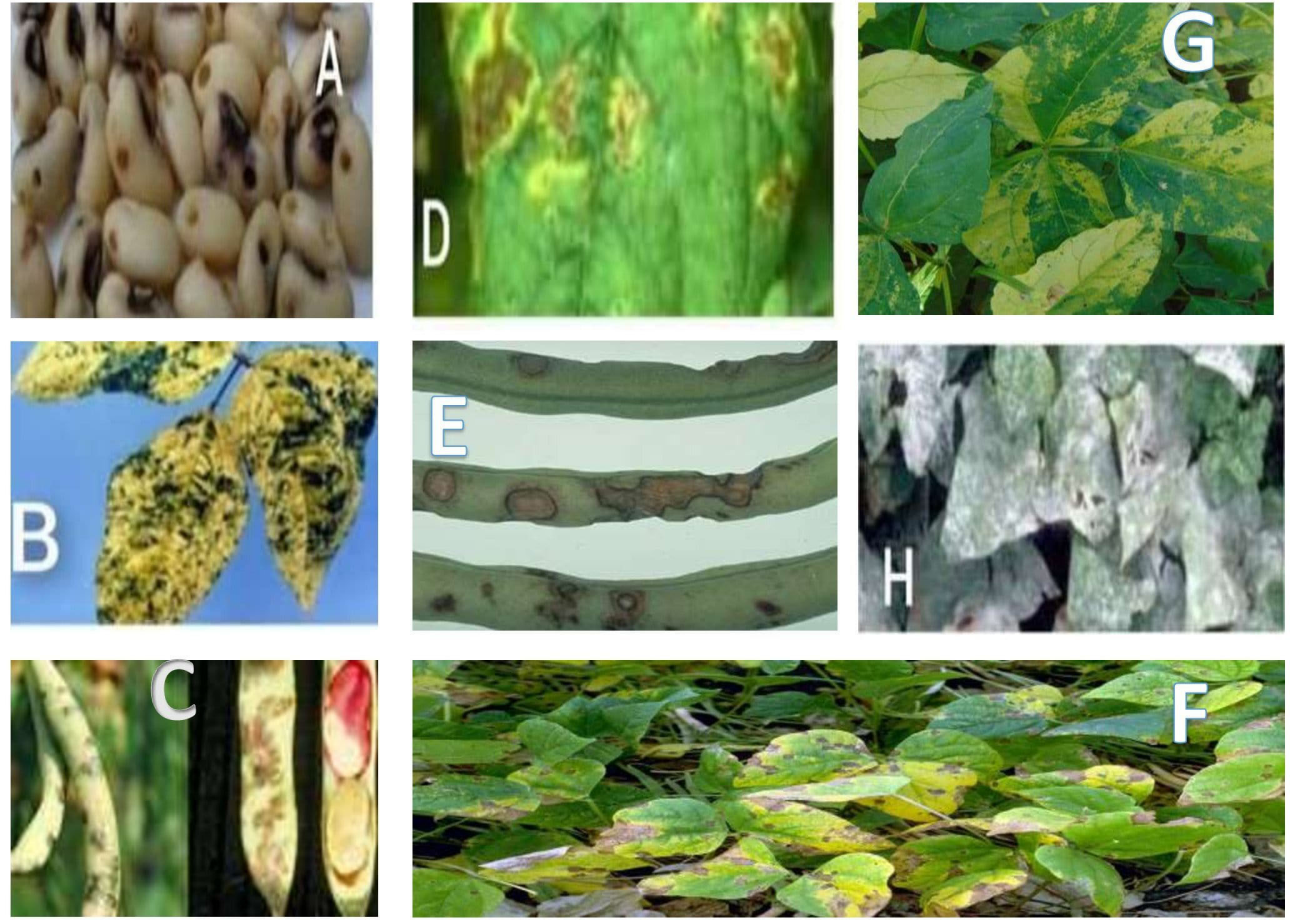
Microbial diseases of cowpea: **(A)** cowpea seed beetle, **(B)** Yellow mosaic virus infected cowpea, **(C)** cowpea halo blight, **(D)** bacterial blight, **(E)** anthracnose, **(F)** cowpea cercospora leaf spot, **(G)** cowpea severe mosaic virus, **(H)** powdery mildew.

The authors apologize for this error and state that this does not change the scientific conclusions of the article in any way. The original article has been updated.

## Publisher’s note

All claims expressed in this article are solely those of the authors and do not necessarily represent those of their affiliated organizations, or those of the publisher, the editors and the reviewers. Any product that may be evaluated in this article, or claim that may be made by its manufacturer, is not guaranteed or endorsed by the publisher.

